# Enhancing Therapeutic Response and Overcoming Resistance to Checkpoint Inhibitors in Ovarian Cancer through Cell Cycle Regulation

**DOI:** 10.3390/ijms251810018

**Published:** 2024-09-17

**Authors:** Shiqi Wang, Chenggui Luo, Jiaqing Guo, Rui Hu, Binglin Shen, Fangrui Lin, Chenshuang Zhang, Changrui Liao, Jun He, Yiping Wang, Junle Qu, Liwei Liu

**Affiliations:** State Key Laboratory of Radio Frequency Heterogeneous Integration, Key Laboratory of Optoelectronic Devices and Systems of Guangdong Province and Ministry of Education, College of Physics and Optoelectronic Engineering, Shenzhen University, Shenzhen 518060, China; wangshiqi20181@email.szu.edu.cn (S.W.); 2110456018@email.szu.edu.cn (C.L.); gjq@szu.edu.cn (J.G.); rhu@szu.edu.cn (R.H.); shenblin@zju.edu.cn (B.S.); lfr1993@163.com (F.L.); chshuzhang@163.com (C.Z.); cliao@szu.edu.cn (C.L.); hejun07@szu.edu.cn (J.H.); ypwang@szu.edu.cn (Y.W.); jlqu@szu.edu.cn (J.Q.)

**Keywords:** cell cycle, immunotherapy, ovarian cancer, FRET sensor, FLIM

## Abstract

Tumor cells invade normal surrounding tissues through continuous division. In this study, we hypothesized that cell cycle regulation changes the immune efficacy of ovarian cancer. To investigate this hypothesis, a Förster resonance energy transfer (FRET) sensor was constructed to characterize the cell activity in real time. Cell shrinkage caused by apoptosis induces the aggregation of proteins on the cell membrane, leading to variations in the fluorescence lifetime of FRET sensors. Moreover, we tracked cell activity across various cycles following co-culture with an immune checkpoint inhibitor. Consequently, we assessed how cell cycle regulation influences immunotherapy in a tumor mouse model. This approach, which involves inhibiting typical cell cycle processes, markedly enhances the effectiveness of immunotherapy. Our findings suggest that modulating the cycle progression of cancer cells may represent a promising approach to enhance the immune response of ovarian cancer cells and the efficacy of immunotherapy based on immune checkpoint inhibitors.

## 1. Introduction

Ovarian cancer, characterized as a malignant tumor, exhibits the highest mortality rate among gynecological cancers [1,2]. Patients with ovarian cancer still have a low five-year survival rate. Complex interactions between cell cycle stages and the tumor microenvironment (TME) have been proposed to influence immunotherapy outcomes in cancer [3,4]. A cell cycle is a tightly regulated series of events that occurs in a cell during growth and division and is governed by multiple proteins to prevent continuous and excessive proliferation. Recently, cell cycle checkpoint inhibitors have been used as a new strategy for ovarian cancer treatment [5]. Prexasertib, an inhibitor of cell cycle checkpoint kinases 1 and 2, demonstrated clinical efficacy and was well tolerated in patients with wild-type BRCA high-grade serous ovarian carcinoma [6]. The Phase I/II trial investigated the novel checkpoint kinase 1 (Chk1) inhibitor SRA737 in combination with gemcitabine, demonstrating tumor responses in patients with ovarian cancer [7]. Consequently, the integration of cell cycle regulation and immune checkpoint inhibitors presents a viable approach to enhance survival rates in ovarian cancer treatment.

Checkpoint inhibitors that achieve targeted immune treatment are anticipated to emerge as a novel approach for improving the survival of patients with ovarian cancer [8]. Notably, owing to the lack of T lymphocyte infiltration and the inability of T cells to recognize all tumor antigens, merely 10% of patients with ovarian cancer experience a favorable prognosis following sole immunotherapy treatment [9,10]. To explore the influence of different cancer cell cycles on immunotherapy, we assess an approach for monitoring the activity of ovarian cancer cells in real time. It has been reported that the process of apoptosis is accompanied by morphological changes such as reduced size and cell membrane shrinkage [11]. Förster resonance energy transfer (FRET), limited to 10 nm proximity between donor and acceptor molecules, offers high specificity and sensitivity in detecting membrane protein clustering [12,13,14]. Moreover, fluorescence lifetime imaging microscopy (FLIM) has been employed to obtain quantitative information regarding FRET without being affected by photobleaching [15,16,17]. The use of FLIM-FRET is expected to enable real-time monitoring of cell status.

Cell cycle regulation is crucial for normal organism growth, yet the impact of the cell cycle on in vivo immune checkpoint therapy remains unexplored. In this study, we demonstrate the influence of cell cycle regulation on immunotherapy for ovarian cancer using a co-culture model, which is not possible with the use of traditional apoptotic indicators. Additionally, we assess the impact of cell regulation on immunotherapy in a mouse model while analyzing redox changes within tumor tissues. Collectively, we demonstrate the utility of the FRET sensor in monitoring cell activity and illustrate that modulating ovarian cancer cells to a non-proliferating state can improve the efficacy of immune checkpoint inhibitors and provide a new strategy for the clinical treatment of ovarian cancer.

## 2. Results

### 2.1. FRET Sensor Characterization of Cell Activity

**Figure 1:**
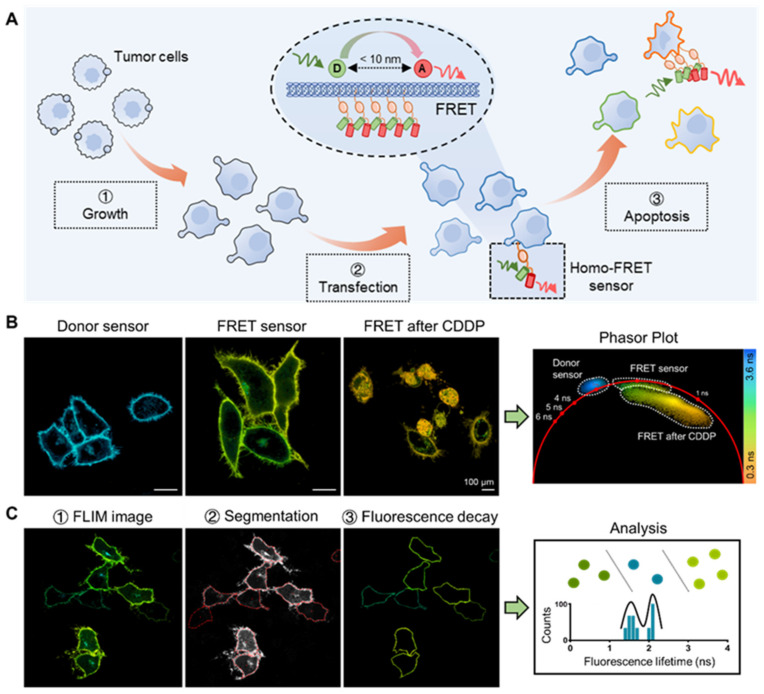
The sensitivity of the FRET sensor to apoptosis induced by immune response. (**A**) The FRET sensor enables the characterization of changes in protein density associated with apoptosis by detecting donor fluorescence lifetime. (**B**) Average lifetime images and the corresponding phasor plot of the cells expressing Lck-V or Lck-Vm, alongside the cells exhibiting FRET sensor activation during chemotherapy. The scale bar is 100 μm. The lifetime values were assigned pseudocolors based on the color scale. (**C**) Cytomembrane segmentation accurately analyzed fluorescence lifetime figures in transfected cells, revealing a distribution of quantities across different donor lifetime intervals at the single-cell level. The fluorescence lifetime of individual cells is shown as color spots in different lifetime intervals.

Apoptosis is a programmed process of cell death characterized by cell shrinkage, chromatin condensation, and fragmentation into small membrane-bound apoptotic bodies [7,18]. In our experiments, we hypothesized that real-time characterization of the cell cycle could be achieved by observing changes in the spatial distribution of membrane proteins resulting from membrane deformation. To verify this hypothesis, we selected Lck as the target protein and constructed a homo-FRET sensor, named Lck-Vm, with Venus and mCherry as the donor and acceptor fluorophores, respectively. Lck is a membrane protein that plays crucial roles in various cellular signal transduction pathways [19,20,21]. Changes in the distance between adjacent proteins after sensor expression affected the FRET efficiency, causing variations in the fluorescence lifetime. These changes were observable as shifts in the cell membrane color on the fluorescence lifetime color map (Figure 1A). For comparison, we generated a gene expression vector that possessed an identical structure solely fused with Venus, named Lck-V.

To prove the above conjecture, we added Cis-diamminedichloroplatinum(II) (CDDP) to the Lck-V- and Lck-Vm-transfected ovarian cancer cells and selected a 525/50 nm bandpass filter to obtain the fluorescence lifetime image of the cells before and after chemotherapy (Figure 1B). The results show no significant change in the fluorescence lifetime of the ovarian cancer cells transfected with Lck-V before and after chemotherapy, and the distribution is 3 ns (Figure 1B). The fluorescence lifetime of Lck-Vm-transfected ovarian cancer cells was significantly changed following chemotherapy, ranging from 0.9–2.8 ns to 0.7–2 ns. The distribution in the fluorescence lifetime across the experimental group and control group can be seen more directly through the phasor plot (Figure 1B). In addition, we optimized the data analysis process to obtain fluorescence lifetime distributions on individual cells. The outcomes of the global analysis are influenced by fluorescence signals present in the cytoplasm generated by liposome transfection (Figure 1C). Therefore, the cytomembrane segmentation method was employed to enhance the precision of lifetime expectancy outcomes. Based on the proposed approach, we conclude that activation of the apoptosis signaling pathway reduces Lck protein spacing, which is manifested by a shortened fluorescence lifetime of the donor.

### 2.2. Immune Checkpoint Therapy Drives Apoptosis of Tumor Cells in Co-Culture Model

**Figure 2:**
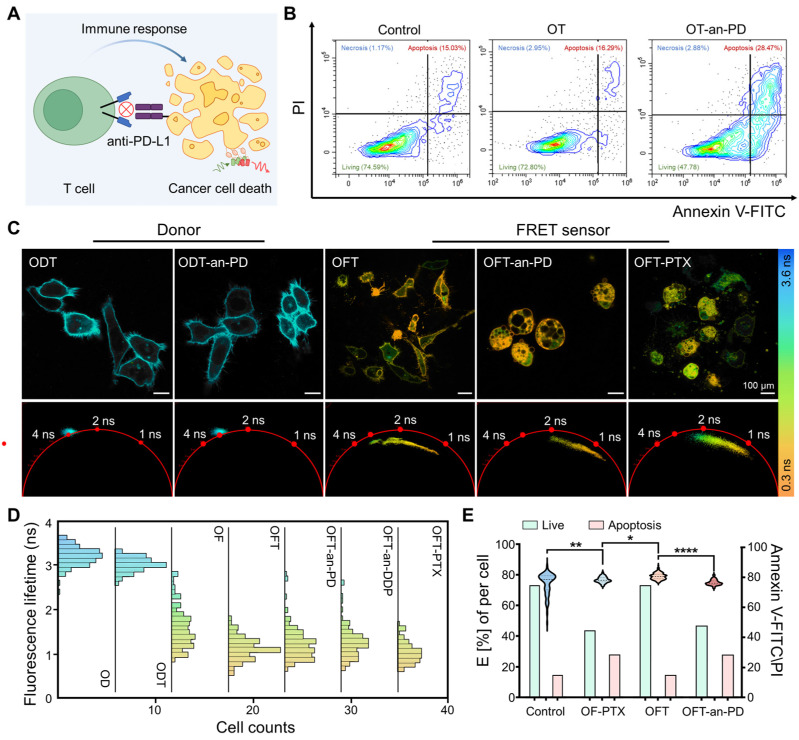
Apoptosis of cancer cells induced by immunotherapy or chemotherapy. (**A**) Anti-PD-L1 enhances the recognition of tumor cells by T cells, leading to tumor cell apoptosis. (**B**) The flow analysis unveiled the apoptosis ratio of OVCAR-3 cells across the experimental groups. (**C**) Fluorescence lifetime images were acquired from the OVCAR-3 cells expressing Lck-V and co-cultured with T cells in the presence of a complete culture medium or anti-PD-L1 (left). The fluorescence lifetime images obtained from the Lck-Vm-transfected cancer cells co-cultured with T cells in the presence of a complete culture medium, anti-PD-L1, or PTX (right). The corresponding phasor plot images are depicted. Lifetime values are shown using pseudocolors based on the color scale ranging from 0.3 ns to 3.6 ns. The scale bar is 100 μm. (**D**) The fluorescence lifetime distribution of (**C**). (**E**) The FRET efficiency of (**C**) and the analysis of (**B**). ODT: OVCAR-3 cells only transfected with the donor and co-cultured with T cells, OFT: OVCAR-3 cells transfected with FRET pairs and co-cultured with T cells, and OFT-an-PD: OVCAR-3 cells transfected with FRET pairs co-cultured with T cells, followed by anti-PD-L1 treatment. A total of over 250 cells from 30 images were individually analyzed, and their lifespan results were statistically evaluated. Statistical significance is indicated by *p* < 0.05 (*), *p* < 0.01 (**) and *p* < 0.0001 (****).

Lck-Vm can serve as a cell status indicator to monitor the physiological state of cells during tumor treatment and to study the influence of different cell cycles on immune efficacy (Figure 2A). Therefore, we first constructed a model in which ovarian cancer cells were co-cultured with T cells. We activated lymphocytes extracted from human blood and co-cultured them with human ovarian cancer cells in the same dish (OT group). Subsequently, anti-PD-L1 was added to the co-culture model for treatment (OT-an-PD group), and the dish with only tumor cells was set as the control group (Control). Cancer cell apoptosis was validated using flow cytometry (Figure 2B). Following identical cell manipulation in all groups, the Annexin V-FITC Kit was used to evaluate the apoptosis of OVCAR-3 cells. We employed centrifugation at varying speeds to selectively isolate the tumor cells and T cells by exploiting the disparity in cellular volume [22]. Compared to the control group, cancer cells in the untreated group exhibited a higher apoptosis ratio, increasing from 15.03% to 16.29%. In the immune group, a significant increase (28.47%) in the proportion of apoptotic cells was observed.

The activity of the ovarian cancer cells was characterized using Lck-Vm. OVCAR-3 cells transfected with Lck-V and co-cultured with T cells were used as the control group (ODT). The anti-PD-L1 antibody did not significantly alter the fluorescence lifetime of the cell membrane (ODT-an-PD). The lifetime distributions of ODT and ODT-an-PD were approximately 3 ns (Figure 2C, left). In the experimental group, we initially evaluated the lifetime using a co-culture model in which T cells and the FRET sensor expressing the OVCAR-3 cells were incubated together (OFT). Compared to ODT, a significant change in the OFT lifetime distribution ranging from 0.9 ns to 2.8 ns was observed, with a peak at 1.4 ns (Figure 2C right). Following the addition of anti-PD-L1, the lifetime shifted to a range of 0.7–1.5 ns, with a peak at 1 ns (OFT-an-PD). Furthermore, paclitaxel was added to OFT in the chemotherapy group (OFT-PTX group). In this group, the fluorescence lifetime exhibited a distribution ranging from 0.7 ns to 1.5 ns, accompanied by a peak at 1.1 ns. Using the previously mentioned single-cell lifetime counting method, we performed statistical analysis of the lifetime of each cell, as shown in Figure 2D,E. Previous research has shown that the color mapping corresponds to fluorescence lifetime, which reflects the activity of transfected OVCAR-3 cells. From cyan to orange, the fluorescence lifetime gradually decreases, indicating the transition of cell status from normal to apoptotic. By analyzing the fluorescence lifetime ranges of individual cells within different groups, it can be observed that the fluorescence lifetime of ovarian cancer cells not co-cultured with T cells is relatively evenly distributed. After co-culturing with T cells, the fluorescence lifetime of ovarian cancer cells rapidly converges in the lower lifetime region. Figure 2E presents the statistical analysis of the flow cytometry results, which are compared alongside the fluorescence lifetime data for a comprehensive evaluation. The results demonstrate that the fluorescence lifetime of the ovarian cancer cells decreased after treatment, indicating that both immunotherapy and chemotherapy promoted the apoptosis of the ovarian cancer cells. This finding is consistent with the changes observed in the flow cytometry results, where the treatment accelerated the apoptotic process in ovarian cancer cells.

### 2.3. Dormant or Quiescent Tumor Cells Escape Immune Attack

**Figure 3:**
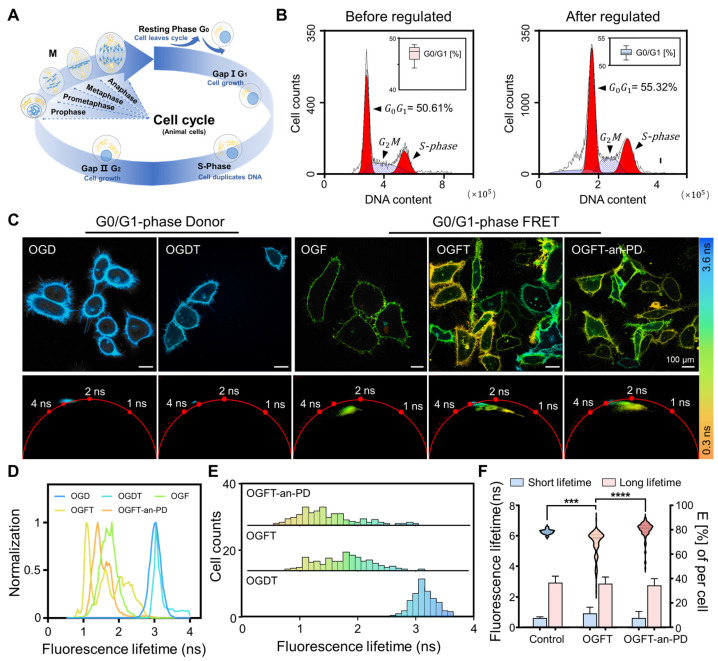
Unveiling the susceptibility of dormant ovarian cancer cells to T cell-mediated immune surveillance by anti-PD-L1. (**A**) Ovarian cancer cells undergo a quiescent phase upon serum deprivation. (**B**) Flow cytometry reveals a shift in the proportion of quiescent cells. (**C**) FLIM images were acquired from OVCAR-3 cells expressing Lck-V or Lck-Vm under different culture conditions, including a complete medium, or co-cultured with T cells in the presence of a complete culture medium or anti-PD-L1. The corresponding phasor plot and the pseudocolor range spanning from 0.3 ns to 3.6 ns are also shown. The scale bar is 100 μm. (**D**) The fluorescence lifetime distribution of (**B**). (**E**) The fluorescence lifetime distribution and (**F**) FRET efficiency of each group after statistics. OGD: regulated OVCAR-3 cells only transfected with donor, OGFT: regulated OVCAR-3 cells transfected with FRET pairs and co-cultured with T cells, and OGFT-an-PD: regulated OVCAR-3 cells transfected with FRET pairs co-cultured with T cells, followed by anti-PD-L1 treatment. Over 250 cells from 30 images were individually analyzed, and the lifespan results were statistically assessed. *p* < 0.001 (***) and *p* < 0.0001 (****) represents a highly significant value.

Dysregulation of cell cycle control, particularly during the G1-S phase transition, has been implicated in the pathogenesis of epithelial ovarian cancer (EOC) [23,24]. We investigated the role of the cell cycle in ovarian cancer immunotherapy by modulating cell cycle distribution in ovarian cancer cells in a co-culture model. Ovarian cancer cells entered the G0/G1 phase following serum starvation (Figure 3A) [25]. The flow cytometry results indicate an increase in the proportion of G0/G1-phase cells from 50.61% to 55.32% after cell cycle regulation (Figure 3B). Subsequently, the fluorescence lifetimes of the regulated cells transfected with Lck-V or Lck-Vm were compared under various culture conditions (Figure 3C). Regardless of co-culturing with T cells, the regulated OVCAR-3 cells transfected with Lck-V exhibited a lifetime of approximately 3.1 ns. This result is consistent with that of the unregulated cells treated under the same conditions.

Compared to the control group, the fluorescence lifetime distribution of the co-culture model exhibited a lifetime range of 0.8–3.1 ns, with a peak value of 1.8 ns (OGFT). This trend was different from that observed in the unregulated cell co-culture model (Figure 2C). This is because ovarian cancer cells in the G0/G1 phase exhibit a dormant or quiescent state, displaying low proliferative activity, which in turn makes them less susceptible to the immune response. As illustrated in Figure 3D, following the addition of immune checkpoint inhibitors, the overall fluorescence lifetime of the ovarian cancer cell membrane is reduced, although the difference is less pronounced than prior to the treatment. Furthermore, as illustrated in Figure 3E,F, there is a significant reduction in the fluorescence lifetime, with double peak values at 1.1 ns and 1.4 ns, accompanied by an increase in the FRET efficiency (OGT-an-PD). These results demonstrate that the addition of anti-PD-L1 stimulated the immune response, thus resulting in the recognition and attack of regulated OVCAR-3 cells by immune cells. Although the anti-PD-L1 antibody can activate immune responses and induce apoptosis in OVCAR-3 cells, its treatment outcome falls short of that observed in unregulated ovarian cancer cells (OVCAR3).

### 2.4. Modulating the Cell Cycle Enhances T Cell Activation In Vivo

**Figure 4:**
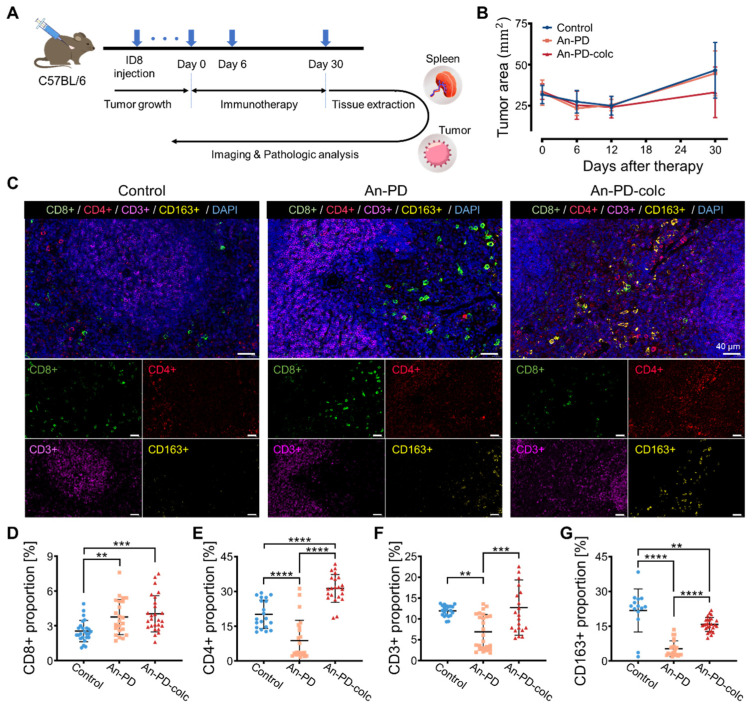
Tumor inhibition in vivo by colchicine in combination with anti-PD-L1. (**A**) A flow chart depicting the immunotherapeutic approach in a murine tumor model. (**B**) The combination treatment group exhibited significantly reduced tumor size. (**C**) Immunofluorescent staining was performed to detect the presence of CD^8+^, CD^4+^, CD^3+^, and CD^163+^ immune cells in the spleen tissue. DAPI was used for nuclear staining. The scale bar is 40 μm. (**D**–**G**) The cell proportions were compared among different experimental groups. Fluorescence expression in over 15 ROI areas was quantified. *p* < 0.01 (**), *p* < 0.001 (***) and *p* < 0.0001 (****) denotes a highly significant difference.

To further evaluate the effect of cell cycle regulation on in vivo immunotherapy, we constructed a mouse ovarian cancer tumor model. Small ovarian tumors that appeared six weeks after ID8 injection were subjected to a five-week treatment regimen involving anti-PD-L1 alone or in combination with colchicine (Figure 4A). The tumor area of the mice was measured weekly during the treatment cycle (Figure 4B). Following euthanasia, the spleen and tumor tissue were sectioned for imaging and pathological analysis.

The presence of T cells and specific subpopulations such as CD^3+^, CD^4+^, and CD^8+^ serves as a strong indicator of an effective antitumor immune response and confers a significant survival advantage [26,27]. High PD-L1 expression is associated with worse five-year and overall survival rates and exhibits a negative correlation with CD^8+^ [28,29]. The expression level of CD^163+^ in malignant lesions is significantly higher than that in benign lesions, which is strongly associated with poor prognosis for ovarian cancer [30,31]. To assess the immune response, cells were immunohistochemically stained for CD^8+^, CD^4+^, CD^3+^, and CD^163+^ in the spleen (Figure 4C). It is evident from the results that the combination treatment upregulated the expression of CD^8+^ (Figure 4D), CD^4+^ (Figure 4E), and CD^3+^ (Figure 4F), while concurrently suppressing the expression of CD^163+^ (Figure 4G). These results suggest that the combination therapy activated the splenic lymphocytes, thereby enhancing the specific immune response in the murine model. In addition, the combination treatment resulted in a significant increase in the number of CD^4+^ and CD^3+^ cells. This implies that the colchicine-induced regulation of the cell cycle effectively increased the number of related immune cells in the spleen and boosted the immune response.

### 2.5. The Tumor Microenvironment Reveals the Clinical Response to Immunotherapy

**Figure 5:**
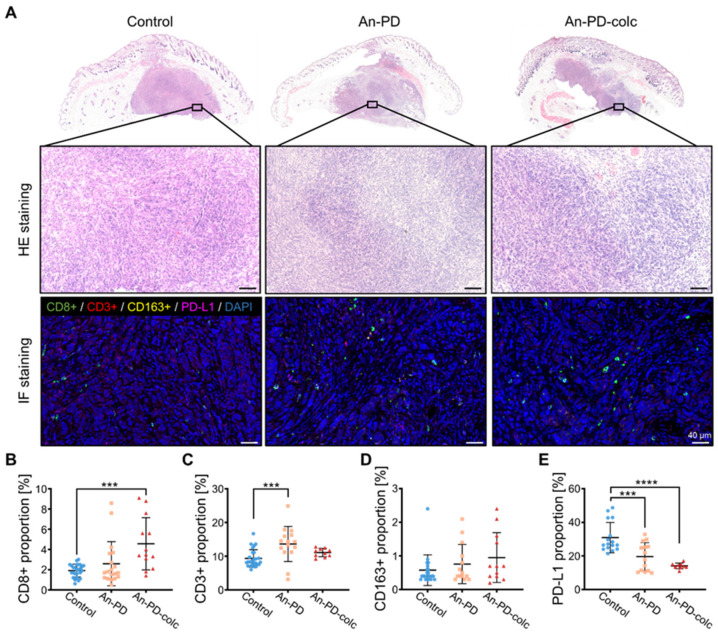
The efficacy of combination therapy was assessed using IF staining. (**A**) Representative images of HE and IF staining for each group. IF staining was performed to detect the presence of CD^8+^, CD^3+^, CD^163+^, and PD-L1 in the tumor tissue. Nuclei are depicted in blue (DAPI). The scale bar is 40 μm. (**B**–**E**) T cell and PD-L1 proportions were compared among different experimental groups. Fluorescence expression across more than 15 ROI regions was measured, and the results were statistically evaluated. *p* < 0.001 (***) and *p* < 0.0001 (****) shows a highly significant result.

To further evaluate the immune efficacy of the mouse ovarian models, tumor tissues were obtained from each experimental group. Hematoxylin–eosin (HE) staining revealed the presence of basal-like cell clusters in all three experimental groups. The stroma of the anti-PD-1-treated tumors appears more mucoid, while the untreated stroma is denser. In the control group, cancer cells infiltrated the dermis, and a significant number of eosinophilic and inflammatory cells were observed at the tumor margin (Figure 5A). Statistical analysis was performed to compare immune cell infiltration between the groups (Figure 5B–E). All treatment groups exhibited a significant upregulation in the proportion of CD^8+^ and CD^3+^ cells compared with the control group. Additionally, there was a significant reduction in the expression of PD-L1 within tumor tissues. It is worth mentioning that the administration of colchicine significantly increased the number of CD^8+^ cells in both the spleen and tumor tissues, whereas no obvious changes were observed in the expression of CD^4+^ and CD^3+^. While the expression of CD^163+^ in the tumor tissues did not demonstrate any significant differences, it was significantly downregulated in the spleen tissues of the treatment group. Immunofluorescence (IF) staining revealed alterations in tumor-infiltrating lymphocytes (TILs) and tumor-associated macrophages (TAMs) following treatment, indicating favorable synergistic therapeutic outcomes.

## 3. Discussion

Investigating the impact of cancer cell cycle distribution on immune checkpoint therapy is crucial for enhancing therapeutic effectiveness in ovarian cancer. In this study, we constructed a FRET sensor capable of targeting membrane proteins to characterize cell activity in a real-time co-culture model for the purpose of exploring our hypothesis that cell cycle regulation influences immunotherapy. Through statistical analysis of the donor fluorescence lifetime on cell-expressed FRET sensors, we determined that the apoptotic process of cancer cells was accompanied by a shortening of the fluorescence lifetime. We speculate that this may be due to the membrane protein clustering caused by apoptosis, which increases the FRET efficiency and decreases the fluorescence lifetime of the donor.

Next, we designed controlled trials to investigate the effects of normal and non-advanced ovarian cancer cells on the efficacy of immunotherapy. We observed that in the co-culture model of the normal ovarian cancer cells and T cells, the addition of anti-PD-L1 did not elicit a significant immune response, which differed from that in the paclitaxel-based treatment group. Subsequently, the ovarian cancer cells were modulated to the G0/G1 phase by serum starvation and co-cultured with the T cells. The results indicated that the addition of anti-PD-L1 enhanced the susceptibility of the T cells to quiescent cancer cells. Moreover, we constructed a mouse ovarian cancer tumor model to investigate the effects of cell cycle regulation on immune checkpoint therapy. We used colchicine in combination with anti-PD-L1 antibodies to regulate the cell cycle for in vivo tumor therapy. Following the combination therapy, a significant decrease in PD-L1 expression and an increase in the number of CD^8+^ cells were observed. Our findings demonstrate that regulation of the cell cycle encourages antibodies to obstruct the PD-L1 pathway, providing a novel opportunity to stimulate T cells at tumor sites to identify and eliminate cancerous cells.

In our research, the observed immune responses and treatment effects are consistent with previously reported outcomes involving checkpoint inhibitors and chemotherapy combinations. However, our results also provide unique insights, particularly in the modulation of PD-L1 expression and immune cell infiltration. Specifically, we observed that treatment not only increased immune cell infiltration, such as CD^8+^ and CD^3+^ T cells, but also dynamically regulated PD-L1 expression within the tumor microenvironment. This suggests a potential feedback mechanism where increased immune activity could upregulate PD-L1 as a countermeasure to evade immune surveillance. Such modulation highlights the complexity of the tumor–immune interaction and the need for strategies that can effectively target both the immune cells and the tumor’s immune evasion mechanisms. Our findings open up avenues for exploring combination therapies that may further enhance immune cell infiltration while counteracting PD-L1-mediated resistance. Additionally, based on our current research, certain improvements can be made. FRET efficiency is highly dependent on the proximity of donor and acceptor molecules, typically within 1–10 nm, which can limit its application in studying larger molecular interactions. Additionally, in complex biological environments, factors such as fluorophore photostability, background fluorescence, and tissue autofluorescence can affect signal sensitivity. In future studies, based on the above limitations, experimental design and probe selection should be improved to enhance the accuracy and sensitivity of FRET experiments in complex biological systems. In summary, our results reveal that cell cycle regulation can improve the therapeutic response and is expected to be a feasible strategy for improving the immune efficacy of ovarian cancer treatments.

## 4. Materials and Methods

### 4.1. Cell Culture

Human ovarian cancer cell line OVCAR-3 (ATCC, Manassas, VA, USA) and mouse ovarian cancer cell line ID8 (ATCC, Manassas, VA, USA) were maintained in Dulbecco’s Modified Eagle’s medium (DMEM) supplemented with 10% fetal bovine serum (FBS) and 1% antibiotic–antimycotic solution (Gibco, Glance, NY, USA). OVCAR-3 cells maintained in the G0 phase were subjected to starvation and cultured in DMEM supplemented with 1% FBS and 1% antibiotic–antimycotic solution. Peripheral Blood Mononuclear cells (PBMCs) were isolated from human blood using Ficoll density gradient centrifugation with Lymphoprep (Thermo Fisher Scientific, Glance, NY, USA). PBMCs were cultured in Roswell Park Memorial Institute (RPMI) medium supplemented with 15% FBS and 1% antibiotic–antimycotic solution. All cells were cultured in 37 °C incubators with 5% CO_2_.

### 4.2. FRET Sensor Constructs and Transfects

The FRET sensor was constructed according to the methodology established by Ma et al. [32]. Venus and mCherry were chosen as the donors and receptors, respectively. The structure of the sensor is illustrated in Figure S1 and was realized by VectorBuilder, Inc. (Guangzhou, China). Following a 24 h incubation period in cell culture dishes (Coherent Scientific, Santa Clara, CA, USA), OVCAR3 cells were transfected using Lipofectamine 3000 (Thermo Fisher Scientific, Glance, NY, USA).

### 4.3. OVCAR-3 Cell/T Cell Coculture

First, 1 × 10^5^ tumor cells were cultured alone for 24 h and transfected with plasmids. For T cell activation, a 5 µg/mL solution of the anti-CD3 (OKT3) antibody (AH003, MultiSciences, Hangzhou, China) was prepared in sterile PBS to coat a 96-well plate, with 50 µL of the antibody or sterile PBS (for controls) added to each well, followed by incubation at 37 °C for 2 h or overnight at 4 °C; after washing twice with PBS, PBMCs were resuspended in complete RPMI medium at a concentration of 10^5^ cells/mL, and 100 µL of the cell suspension was added to each well, followed by the addition of 2 µg/mL of the anti-CD28 antibody (AH028, MultiSciences, Hangzhou, China) and incubation at 37 °C for 3 days in a CO_2_ incubator. Subsequently, the culture dishes were repeatedly cleaned with PBS, and then, 1 × 10^5^ T cells were co-cultured with transfected tumor cells for 12 h for follow-up experiments.

### 4.4. Flow Cytometry

Cell death was analyzed using two kits for detecting the cell cycle and apoptosis (Beyotime Biotechnology, Shanghai, China). The cells (1 × 10^6^) were washed and incubated with Annexin V-FITC and PI (or PI and RNase A) according to the manufacturer’s instructions. The cells were washed and resuspended three times before being analyzed using a flow cytometer (Beckman Coulter Inc., Brea, CA, USA). A total of 10,000 events were acquired per sample and subsequently analyzed using FlowJo software 10.0.7 (TreeStar, Ashland, OR, USA). Statistical results are provided in the Supplementary Materials (Figure S2).

### 4.5. Mice

Animal studies were approved by the Guangdong Medical Laboratory Animal Center (Guangdong, China) and conducted according to the ethical code C202110-01. All mice were housed under specific pathogen-free conditions at Shenzhen University. To assess therapeutic efficacy, four- to five-week-old female C57BL/6 mice were subcutaneously injected with 1 × 10^7^ ID8 cells on the right flank. Tumor size was measured and recorded weekly using a high-precision caliper. Once the tumors reached approximately 50 mm^2^, the mice were divided into groups of six for subsequent treatments. One week after the final treatment, the mice were euthanized according to the IACUC guidelines. Tumor and spleen tissues were excised and processed into paraffin slices for histological analysis and optical imaging.

### 4.6. Therapeutic Strategy

The following reagents were used for cellular experiments. Cisplatin and paclitaxel were purchased from Beyotime Biotechnology (Shanghai, China). InVivoMAb anti-mouse PD-L1 (B7-H1) (clone: 10F.9G2™) and InVivoMAb anti-human PD-L1 (B7-H1) (clone: 29E.2A3™) were purchased from Bioxcell (West Lebanon, Lebanon, NH, USA). Then, 1 × 10^5^ transfected OVCAR-3 cells were treated with CDDP, paclitaxel, or anti-PD-L1 at final concentration of 10 μM, 10 nM, or 5 µg/mL for 2 h. Following treatment, the cells were washed with Phosphate-Buffered Saline (PBS, Beyotime, Shanghai, China) to remove any excess drug, and a fresh medium was added. All treatments were performed under standard cell culture conditions at 37 °C in a humidified atmosphere with 5% CO_2_. The group that received single immunization was treated solely with 200 μg/mouse anti-mouse PD-L1 in the mouse model, while 1 mg/kg colchicine was introduced to the combined treatment group. The control group was injected with an equal volume of normal saline solution. The drug was administered weekly through in situ injections, following which the mice were euthanized and the tissue was extracted after five consecutive doses.

### 4.7. Immunofluorescence Imaging

For immunofluorescence observation of the tumor and spleen tissues, paraffin sections were dewaxed and rehydrated. Subsequently, antigen retrieval was performed using sodium citrate to enhance sample quality. The sections were washed and blocked with 3% bovine serum albumin for 30 min, followed by incubation with primary antibodies overnight at 4 °C and secondary antibodies for 1 h at room temperature. IF staining of the tissue sections was performed using DAPI (Servicebio, Wuhan, China), PD-L1 antibodies (protein tech, Wuhan, China), and various immune cell markers, including CD^8+^ (Servicebio, Wuhan, China), CD^163+^ (Servicebio, Wuhan, China), CD^3+^ (Servicebio, Wuhan, China), and CD^4+^ (Thermo Fisher Scientific, Glance, NY, USA).

### 4.8. Imaging and Analysis

Optical imaging was conducted using a multimodal imaging system based on a femtosecond laser (Chameleon Discovery, Coherent, Santa Clara, CA, USA) with a pulse width of 100 fs and a repetition rate of 80 MHz. A 60 × objective camera (1.4 NA, Nikon) was used for the cell image, and a 20 × objective camera (MRD70200, 0.75 NA, Nikon) was used for the tissue image. For cellular imaging, a combination of 960 nm and BP 525/50 was selected to detect the fluorescence emitted by Venus. The image resolution was set to 1024 × 1024 pixels, with a scanning speed of 0.25 µm/px. Fluorescence lifetimes were measured using a time-correlated single-photon counting (TCSPC) module (SPC-150, Becker & Hickl GmbH, Berlin, Germany). We selected laser wavelengths of 780 nm and 840 nm to excite (Nicotinamide adenine dinucleotide) NADH and (Flavin adenine dinucleotide) FAD, respectively. Two filter combinations were employed to isolate distinct AF signals: (1) BP 460/40 nm for NADH and (2) BP 550/40 nm for FAD. An image resolution of 512 × 512 pixels was selected for all of the FLIM images, and the cumulative number of photons in each pixel was not less than 300. To reduce potential artifacts, we meticulously controlled experimental conditions such as excitation intensity and sample preparation and utilized control samples to ensure accurate and reliable FRET measurements, minimizing photobleaching and other technical interferences. Over 30 distinct regions of interest (ROIs) were randomly identified for each test group. TP-FLIM images were analyzed using maximum likelihood estimation (MLE) with SPCImage software 9.0 (Becker & Hickl GmbH, Berlin, Germany). The E refers to the FRET efficiency of the interacting donor fraction and was calculated as 1 minus the ratio of t_1_ to t_2_, where t_1_ is the donor lifetime in the presence of the acceptor, and t_2_ is the donor lifetime alone.

### 4.9. Statistical Analysis

The fluorescence lifetime of individual cells in each image was quantified, with approximately 200 cells analyzed per group (n ≈ 200). The mean ± standard deviation (s.d.) of all measurement data was calculated from at least three independent experiments and analyzed using GraphPad Prism 9 (La Jolla, San Diego, CA, USA). Statistical significance was determined using the one-way ANOVA method, with significance set at *p* < 0.05 (*), *p* < 0.01 (**), *p* < 0.001 (***) and *p* < 0.0001 (****).

## Data Availability

All data are available in the main text or the Supplementary Materials (Figures S1–S3 and Table S1).

## Supplementary Materials

The following supporting information can be downloaded at: https://www.mdpi.com/article/10.3390/ijms251810018/s1.
