# Supplementary material for: Enhancing Therapeutic Response and Overcoming Resistance to Checkpoint Inhibitors in Ovarian Cancer through Cell Cycle Regulation
Source: Int J Mol Sci. 2024 Sep 17;25(18):10018. doi: 10.3390/ijms251810018 (PMC11431879; doi:10.3390/ijms251810018)
Supplement: Supplementary file 1 [file ijms-25-10018-s001.zip › ijms-3195438-supplementary.pdf]

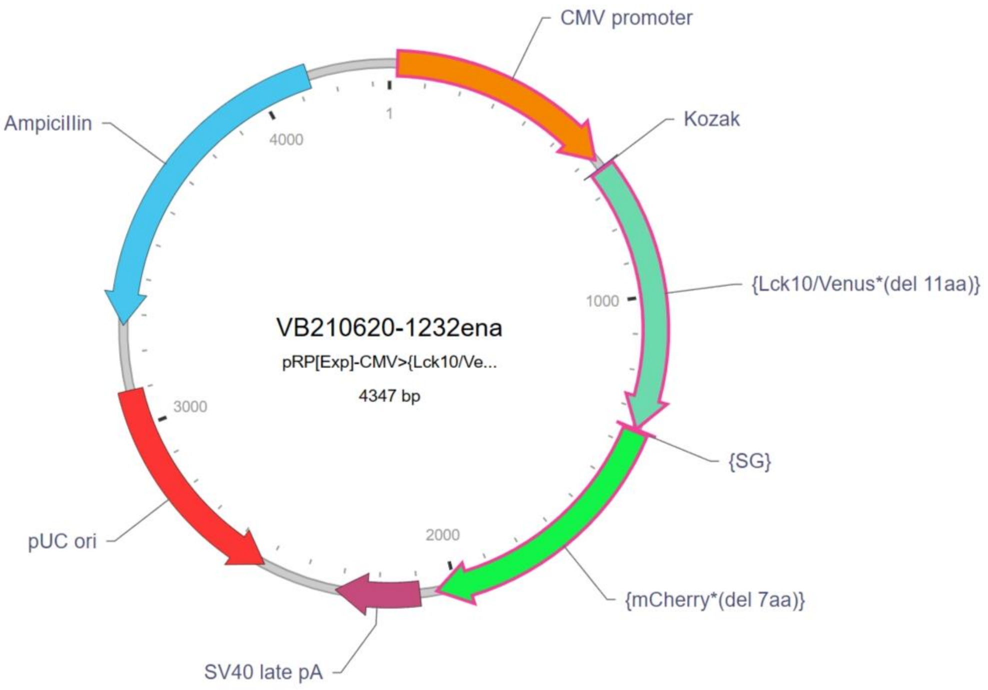


**Figure S1.** Structure of the FRET sensor. The mcherry and Venus were used as donors and recipients of single-strand FRET. The fluorescence lifetime of the donor in the plasmid changes with the shrinkage of the cell membrane.

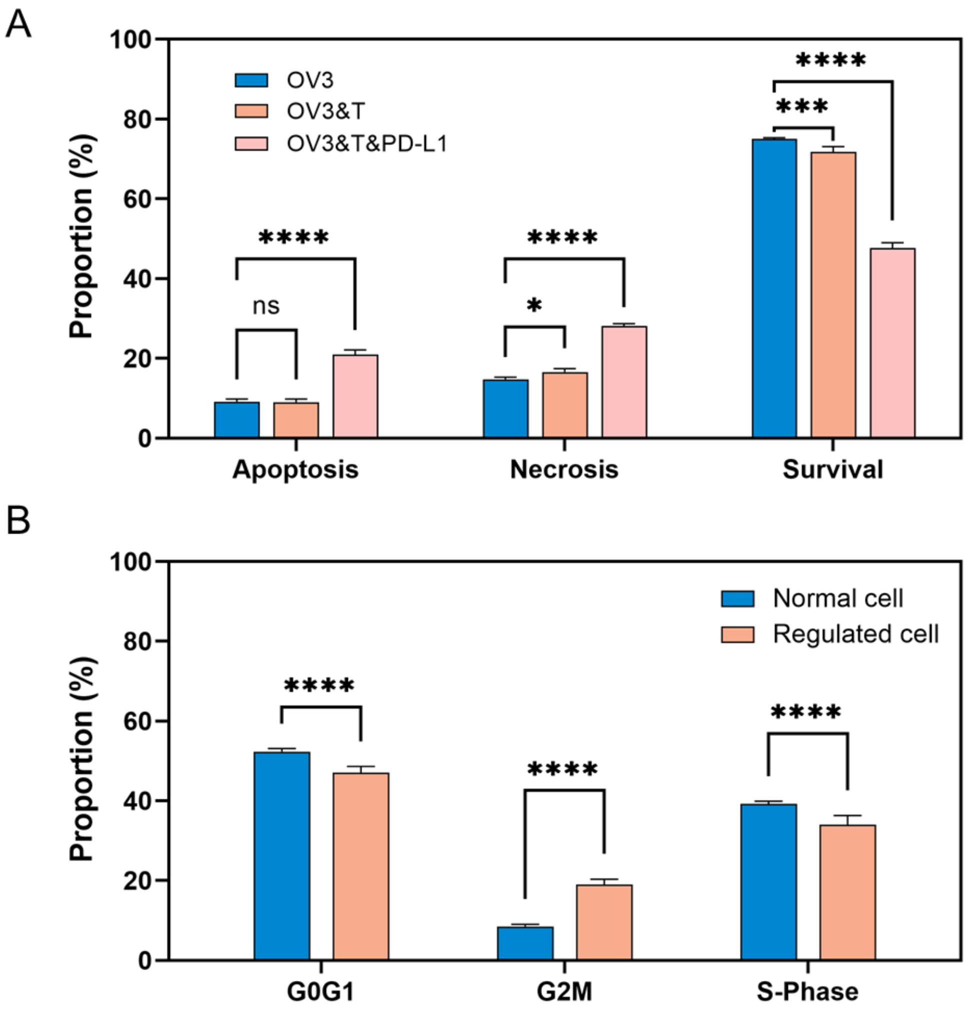


**Figure S2.** (**A**,**B**) Statistical results of Flow cytometry for cell cycle or apoptosis. Annexin V-FITC kit and kit were used to evaluate the apoptosis and cell cycle degree of cancer cells. $p < 0.05$(*), $p < 0.001$ (***) and $p < 0.0001$ (****) denotes a highly significant difference.

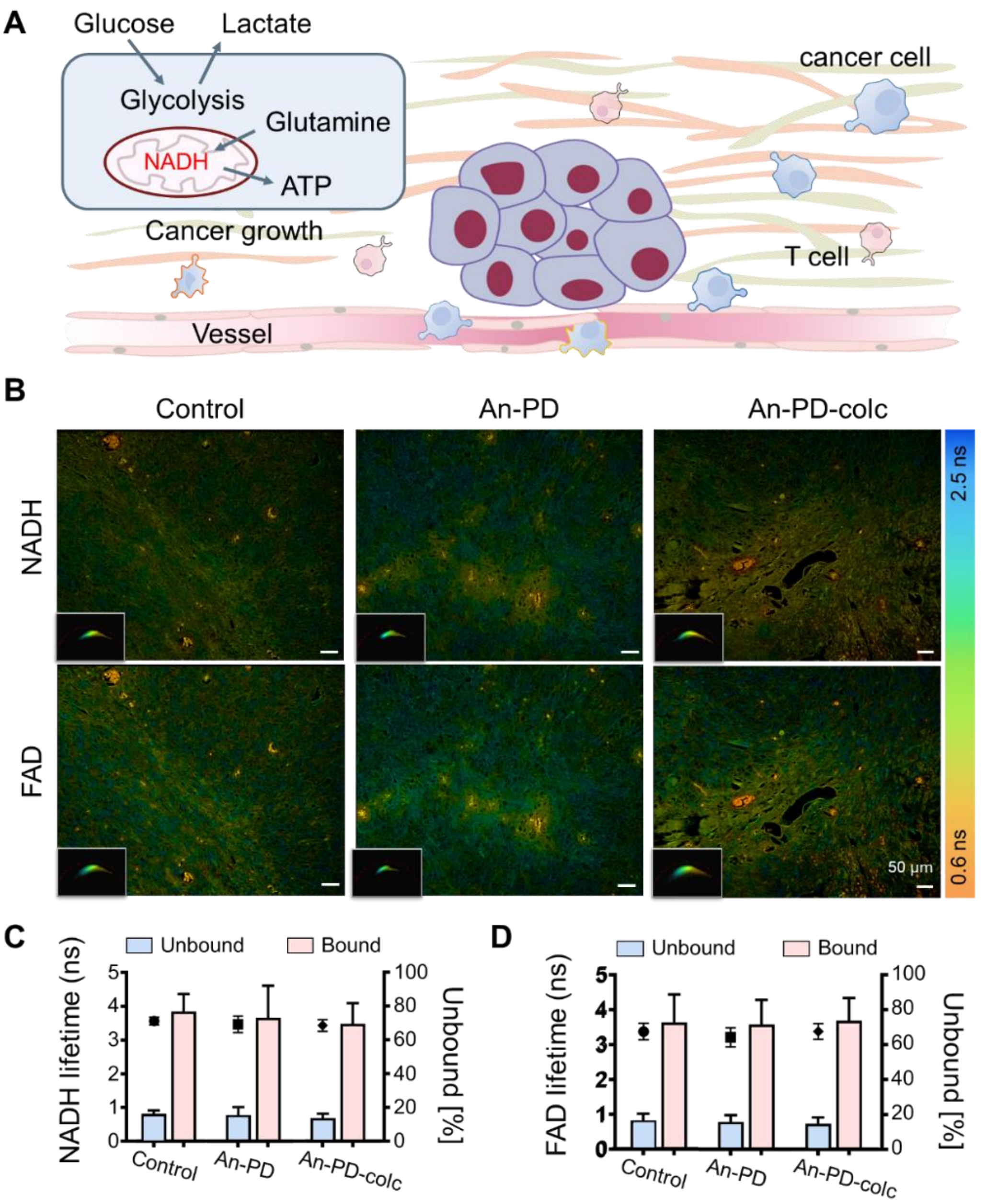


**Figure S3.** The efficacy of combination therapy was assessed using FLIM. (**A**) Schematic illustration of the tumor microenvironment. The tumor growth-induced changes in the reduction-oxidation ratio are manifested as alterations in fluorescence lifetime. (**B**) and (**C**) A bi-exponential decay model effectively characterized the NADH and FAD fluorescence decays observed across different treatment groups. (**D**) Lifetime images of all groups and the insertion graphs are the corresponding phasor plots. The slice thickness is 4 μm. Scale bar is 50 μm.

FLIM imaging was performed on all groups to characterize the changes in the tumor microenvironment. According to the Warburg effect, tumor cells utilize glycolysis to absorb large amounts of glucose from microenvironments meeting their rapid growth demands (Figure S3A). When the metabolic process shifts from oxidative phosphorylation to glycolysis, the apparent lifetime of Nicotinamide adenine dinucleotide (NADH) decreases, whereas that of Flavin adenine dinucleotide (FAD) increases. The FAD content in the slices following the combined treatment exhibited a decreasing trend compared with that in the other two experimental groups (Figures S3B and S3C). This suggests that the level of glycolysis in the tumor region was reduced in the combined treatment group, which further indicates the decreased activity of the tumor cells.

**Table S1. Abbreviation list.**

| | |
|---|---|
| FRET | Förster resonance energy transfer |
| TME | Tumor microenvironment |
| FLIM | Fluorescence lifetime imaging microscopy |
| EOC | Epithelial ovarian cancer |
| HE | Hematoxylin-eosin |
| IF | Immunofluorescence |
| TILs | Tumor-infiltrating lymphocytes |
| TAMs | Tumor-associated macrophages |
| DMEM | Dulbecco's modified eagle's medium |
| FBS | Fetal bovine serum |
| PBMCs | Peripheral blood mononuclear cells |
| RPMI | Roswell Park Memorial Institute |
| TCSPC | Time-correlated single-photon counting |
| NADH | Nicotinamide adenine dinucleotide |
| FAD | Flavin adenine dinucleotide |
| ROIs | Regions of interest |
| MLE | Maximum likelihood estimation |
